# Collective effects of cell cleavage dynamics

**DOI:** 10.3389/fcell.2024.1358971

**Published:** 2024-03-15

**Authors:** Magdalena Schindler-Johnson, Nicoletta I. Petridou

**Affiliations:** ^1^ Developmental Biology Unit, European Molecular Biology Laboratory, Heidelberg, Germany; ^2^ Faculty of Biosciences, Heidelberg University, Heidelberg, Germany

**Keywords:** cell cleavage, embryogenesis, cell cycle, tissue morphogenesis, collective behavior and dynamics, synchrony, variability

## Abstract

A conserved process of early embryonic development in metazoans is the reductive cell divisions following oocyte fertilization, termed cell cleavages. Cell cleavage cycles usually start synchronously, lengthen differentially between the embryonic cells becoming asynchronous, and cease before major morphogenetic events, such as germ layer formation and gastrulation. Despite exhibiting species-specific characteristics, the regulation of cell cleavage dynamics comes down to common controllers acting mostly at the single cell/nucleus level, such as nucleus-to-cytoplasmic ratio and zygotic genome activation. Remarkably, recent work has linked cell cleavage dynamics to the emergence of collective behavior during embryogenesis, including pattern formation and changes in embryo-scale mechanics, raising the question how single-cell controllers coordinate embryo-scale processes. In this review, we summarize studies across species where an association between cell cleavages and collective behavior was made, discuss the underlying mechanisms, and propose that cell-to-cell variability in cell cleavage dynamics can serve as a mechanism of long-range coordination in developing embryos.

## 1 Introduction: cell cleavage dynamics in metazoan development

Cell cleavages constitute the very first divisions of embryo development that proceed without significant cell growth, producing the cell mass required for embryogenesis (Gilbert, 1985). In multicellular embryos (e.g., amphibians and teleosts), in every cleavage round the daughter cells have half the size of the mother cell, whereas in multinucleated systems (e.g., insects) the number of nuclei multiply within a confined space, creating a syncytium (Gilbert, 1985). In many species, the first mitotic divisions are quick, where the cell cycle oscillates between DNA synthesis and mitosis phases with weak or no checkpoints (Raff and Glover, 1988; 1989; Glover, 1989; Newport and Dasso, 1989; Sibon et al., 1997; Zhang et al., 2014). Then, they slow down over time by incorporating gap phases and cell cycle checkpoints, as the system approaches developmental milestones, including gastrulation, cellularization and tissue spreading (Newport and Kirschner, 1982; Foe et al., 1993; Kimmel et al., 1995; Lecuit and Wieschaus, 2000; O’Farrell et al., 2004; Farrell and O’Farrell, 2014). The fast embryonic cycles have been observed in representatives of the major phyla in the evolutionary tree of the metazoa: Mollusca (clam), Arthropoda (fruit fly), Annelida (leeches), Echinodermata (sea urchin and starfish), Chordata (frogs, fish, ascidians, chick), and Nematoda (*Caenorhabditis elegans*) (Eyal-Giladi and Kochav, 1976; Parisi et al., 1978; Nishida, 1987; Bissen and Weisblat, 1989; Wright and Schatten, 1990; Hunt et al., 1992). Even in mammals (mice and rats), where early divisions do not exhibit all the above characteristics, at the crucial moment of gastrulation, they display fast reductive cycles without checkpoints (Snow, 1977; Mac Auley et al., 1993; Fulka et al., 1999; Heyer et al., 2000; O’Farrell et al., 2004; Zernicka-Goetz, 2005), suggesting that the embryonic cell cleavage dynamic pattern does not only give rise to the appropriate cell number, but it may act as a developmental checkpoint or timer of gastrulation.

The spatial cell cleavage pattern is defined by the temporal dynamics of the mitotic divisions, which also exhibit strong similarities between species. Usually the rapid cell divisions are highly synchronous between the cells or nuclei, whereas during the slowing down they start desynchronizing, exhibiting meta-synchronous divisions, and eventually become asynchronous before gastrulation (Satoh, 1977; Newport and Kirschner, 1982; Boterenbrood et al., 1983; Foe et al., 1993; Masui and Wang, 1998; Keller et al., 2008; Olivier et al., 2010; Mendieta-Serrano et al., 2013). The elongation of the cell cycle is observed at the mid-blastula transition (MBT), during which the maternally supplied cell cycle regulators run out and the embryo starts synthesizing its own resources (Langley et al., 2014). Since during meta-synchrony and asynchrony not all regions of the embryo divide at the same time, spatial patterns of mitotic activity are generated, which are often linked to spatial patterns in cell behaviors, such as cortical actomyosin contraction (Rankin and Kirschner, 1997; Chang and Ferrell, 2013; Bischof et al., 2017; Deneke et al., 2019; Shamipour et al., 2019), differential transcription and fate specification (Edgar et al., 1994; Momen-Roknabadi et al., 2016; Ogura and Sasakura, 2016; Anderson et al., 2017; Fabrèges et al., 2023), and changes in tissue mechanical properties (Petridou et al., 2019; Petridou et al., 2021; Fabrèges et al., 2023). Given that the temporal coordination of cell cleavages defines embryonic patterns, it has been thus a long-standing goal to identify the mechanisms regulating mitotic (de)synchronization during embryo development.

**FIGURE 1:**
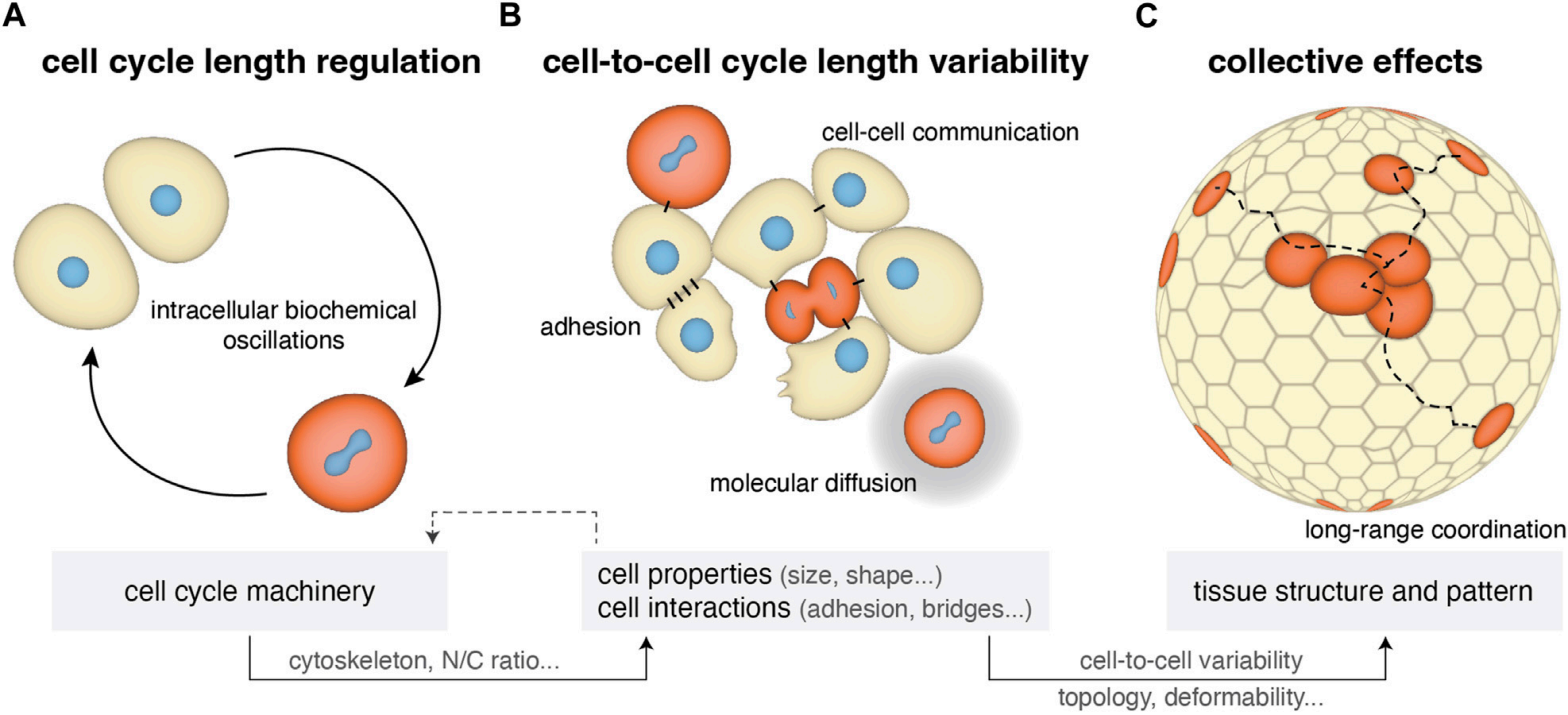
Cell cycle length regulation from single cells to tissues in early cleaving embryos. **(A)** Single-cell cycle length is regulated by internal biochemical processes including CDK/cyclins oscillations, N/C ratio, zygotic genome activation and maternal mRNA regulation (Section 2.1). At the same time it impacts cell physical properties such as cytoskeletal mechanics and cell size. **(B)** Within a cell population the cell’s microenvironment can influence cell cycle length depending on the form of cell-cell communication (cytoplasmic bridges and diffusion of the cell cycle oscillators) and on changes in cell adhesion and cell shape occurring during mitotic rounding, resulting in variability in cell-to-cell cycle lengths (Section 2.2). **(C)** This cell cycle variability can generate further variability in other cell properties (cell size, shape) and impact collective tissue properties, such as topology and deformability (Section 2.3).

The relative cell cycle synchrony (synchronicity) in a population is the outcome of cell cycle regulation within each cell (Figures 1A, B). The length of embryonic cell cycles has been shown to be regulated by the following mechanisms (Ogura and Sasakura, 2017; Liu et al., 2021): oscillatory activity of the cyclin-dependent kinases (Cdks) promoting mitotic entry [reviewed in (Brantley and Di Talia, 2021)], the nucleus-to-cytoplasmic ratio [reviewed in (Balachandra et al., 2022)], and transcriptional/translational mechanisms associated with zygotic genome activation and depletion of maternal gene products that cause cell cycle elongation [reviewed in (Vastenhouw et al., 2019)] (Figure 1A). Although the above mechanisms are regulated locally, at the single cell/nucleus level, they collectively form patterns across the embryo that not only define the spatial profiles of cell divisions, but also of associated cell behaviors (Figure 1C). How such mechanisms affect cell collectives has only recently been started to be addressed.

Here, we first summarize the mechanisms regulating cell cycle elongation in each cell (Section 2.1; Figure 1A) and desynchronization within a cell population (Section 2.2; Figure 1B) during early development. Then, we explore the effects of cell-to-cell synchronicity in cell divisions on collective behavior including embryo-scale physical properties and pattern formation in early embryos (Section 2.3; Figure 1C) and other developing systems (Section 2.4). Finally, we discuss how cell cycle synchronicity, and its degree of variation, may act as a mechanism of information propagation across the embryo and time its transition to an active morphogenetic system.

## 2 Main text

### 2.1 Cellular mechanisms regulating cell cycle length

**FIGURE 2:**
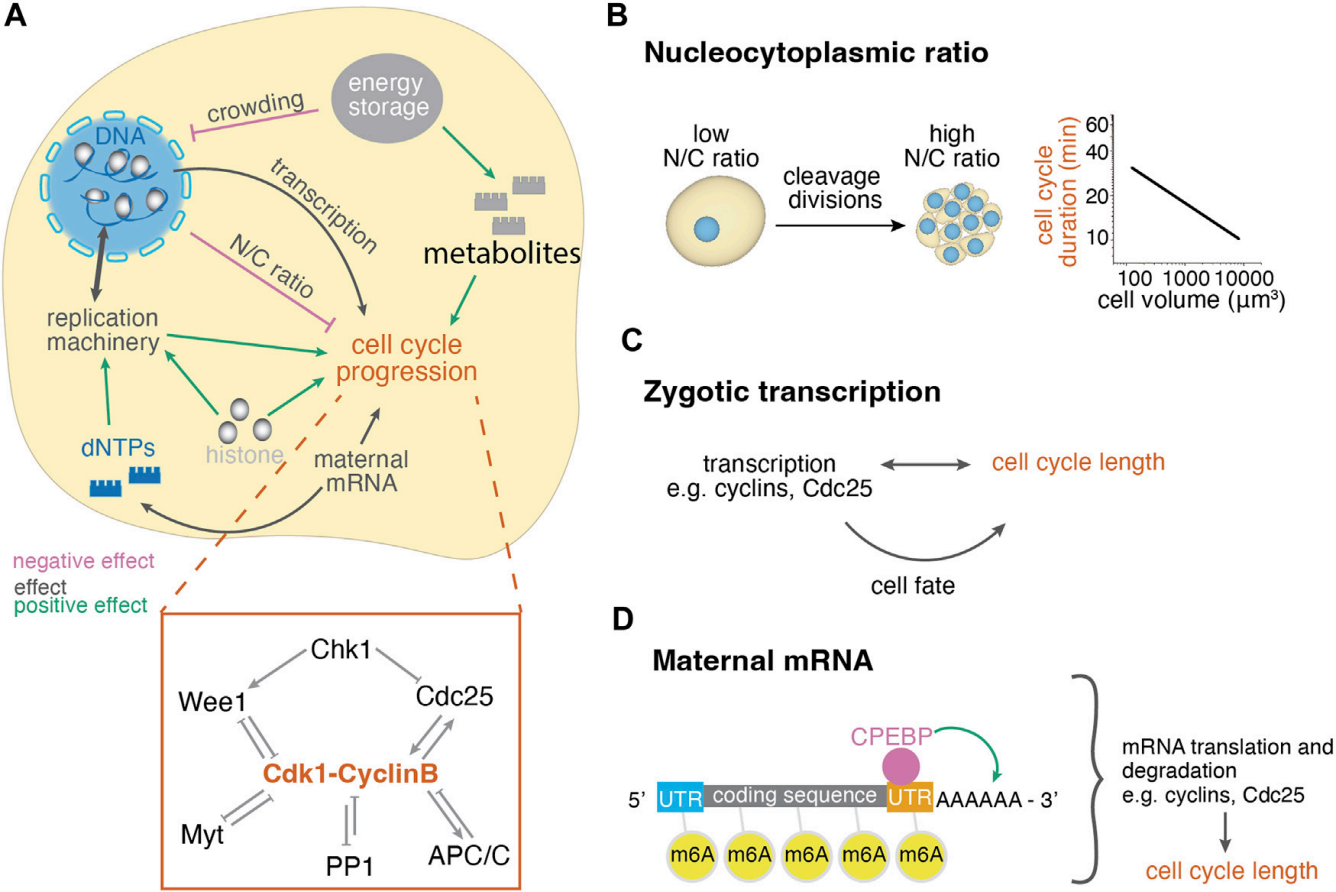
Cellular control mechanisms of cell cycle length changes during embryogenesis. **(A)** Cellular mechanisms regulating the cell cycle length involve the regulation of the concentration of nuclear and cytoplasmic factors and their interplay. Positive effect arrows indicate mechanisms that promote cell cleavages, whereas negative effect arrows indicate mechanisms that delay cell cleavages, thus lengthen the cell cycle. The depicted effects on cell cycle progression have been summarized from various organisms and may not apply for all systems, see text for details. Inset: diagram of mitosis control through Cdk1. Before mitosis, Cdk1 is under inhibition of checkpoint kinase 1 (Chk1) and protein phosphatase 1 (PP1). Mitotic entry is enabled by the Cdk1-cyclinB auto-amplification loop that inhibits its antagonists (Wee1, Myt, PP1) and activates its activator Cdc25. Mitosis exit is enabled by decreased Cdk1-CyclinB activity due to activation of APC/C and PP1. **(B)** The ratio of nuclear to cytoplasmic volume changes throughout cleavage divisions, leading to the titration of cytoplasmic against nuclear components. The right graph shows the negative correlation of cell size and cell cycle length of the AB lineage in early *C. elegans* development (Arata et al., 2014). **(C)** Zygotic transcription may influence the cell cycle directly or indirectly through cell fate while cell cycle length also influences transcriptional potential. **(D)** Maternal mRNA translation and degradation affects the availability of cell cycle regulators and thus the cell cycle length. These processes are regulated through modifications, such as polyadenylation and methylation, by RNA binding proteins.

The mechanisms regulating mitotic synchronicity rely on the regulation of the cell cycle length. The cell cycle length is defined by the components of the cell cycle machinery that control entry and exit to the different cell cycle phases [reviewed in (Sullivan and Morgan, 2007; Heim et al., 2017; Brantley and Di Talia, 2021)]. To regulate the cell cycle, cyclin-dependent kinases (Cdks) bind to cyclins, which fluctuate in their availability throughout the cell cycle. The Cdk1-CyclinB complex promotes the entry to mitosis and its activity depends on the phosphorylation state of Cdk1: inhibitory phosphorylations are placed by Wee1 and Myt and removed by Cdc25 phosphatases (Figure 2A, inset). Once few Cdk1-CyclinB complexes are active, they reinforce their own activity by inhibiting Wee1 and Myt and activating Cdc25, resulting in switch-like activation (Pomerening et al., 2003; Trunnell et al., 2011). Mitosis is triggered after nuclear import of the complex upon phosphorylation of CyclinB, which is also controlled by a bistable switch (Santos et al., 2012). The Cdk1-CyclinB complex inactivates itself by activating the ubiquitin-protein ligase anaphase-promoting complex (APC) to slowly degrade CyclinB (Pines, 2011) and by indirectly activating inhibitory phosphatases, like PP1, promoting thus exit from mitosis (Heim et al., 2017). Furthermore, the DNA damage checkpoint kinase Chk1 can negatively regulate Cdk1-CyclinB activity through Wee1, Cdc25 and through CyclinB translocation (Royou et al., 2008; Patil et al., 2013) (Figure 2A, inset). Cdk1, Cdc25 and Chk1 have been experimentally shown to regulate early embryonic cycles during *Xenopus*, zebrafish, *Drosophila melanogaster* and mouse development (Pomerening et al., 2003; Dalle Nogare et al., 2009; Trunnell et al., 2011; Chang and Ferrell, 2013; Zhang et al., 2014; 2015; Knoblochova et al., 2023). The mechanisms below describe how cell cycle lengthening is achieved *in vivo* by developmentally regulating the components of this universal cell cycle machinery within each cell (Figure 2).

#### 2.1.1 The nucleocytoplasmic ratio

The nucleus-to-cytoplasmic volume (N/C) ratio has been shown to affect cell cycle lengthening during development in many species [reviewed in (Balachandra et al., 2022)]. The reductive nature of early cleavage cycles results in a drastic decrease of cytoplasmic volume while nuclear volume decreases only slightly (Newport and Kirschner, 1982; Kane and Kimmel, 1993; Jevtić and Levy, 2015). Thus, the N/C ratio increases with each cleavage (Figure 2B). Its impact on cell cycle length can be demonstrated by the negative correlation of cell size and cycle length [reviewed in (Arata and Takagi, 2019)] (Figure 2B). The N/C ratio’s importance for cell cycle lengthening was first experimentally demonstrated in *Xenopus* by constriction of the fertilized egg so as only half of the embryo inherits the nucleus. The nucleated side divides until a nucleus moves to the non-nucleated half, triggering its division, however, there it is placed in a larger cytoplasm. In this case, the initially nucleated side desynchronized earlier, and the initially non-nucleated side desynchronized later, but at the same N/C ratio (Newport and Kirschner, 1982). Similar experiments have been performed in newts (Kobayakawa and Kubota, 1981), zebrafish (Kane and Kimmel, 1993), *Drosophila* (Edgar et al., 1986; Deneke et al., 2016) and cricket embryos (Donoughe et al., 2022), suggesting that there is a critical N/C ratio threshold triggering cell cycle lengthening, with an increased or decreased N/C ratio causing earlier or later onset of cell cycle elongation (Balachandra et al., 2022). However, the underlying molecular mechanisms are still under investigation, with work so far identifying changes in the concentration of factors in the nucleus, in the cytoplasm, or in the interactions between the two compartments (Figure 2A). The N/C ratio-dependent cell cycle regulation can display species-dependent characteristics (Farrell and O’Farrell, 2014; Langley et al., 2014; Zhang et al., 2017), which we summarize below.

Within the nucleus, genome size has been demonstrated to affect cell cycle elongation onset in different species. This was shown through ploidy (Newport and Kirschner, 1982; Edgar et al., 1986; Kane and Kimmel, 1993; Jukam et al., 2021) and chromosome-level manipulations (Lu et al., 2009; Blythe and Wieschaus, 2015; Hayden et al., 2022). Histone proteins also affect cell cycle remodeling at MBT, as was observed in *Xenopus*, *Drosophila* and zebrafish (Yue et al., 2013; Amodeo et al., 2015; Chari et al., 2019) (Figure 2A). In zebrafish, it has been suggested that certain maternal histone variants enable rapid cleavage cycles due to a loose chromosomal architecture (Yue et al., 2013). Chromatin remodeling is further enabled by maternally provided complexes such as the NuRD complex, which is required for DNA replication in *Xenopus* and decreases in activity around MBT (Christov et al., 2018). Finally, the size of the nucleus *per se* also contributes to cell cycle length regulation, as was shown in *Xenopus* (Jevtić and Levy, 2015). In this study, nuclear volume was increased by increasing nuclear import (importin-α overexpression) and nuclear surface area (lamin overexpression). Nuclear volume was decreased by decreasing the surface area of the nuclear envelope (overexpression of components of the connected endoplasmic reticulum). These nuclear volume manipulations caused changes of the cell cycle length especially after MBT (Jevtić and Levy, 2015).

The titration of a cytoplasmic pool of maternally deposited factors against the increasing DNA has been the first hypothesis to explain the slowing down of cell cycles during MBT (Newport and Kirschner, 1982). Identified as such factors are replication factors (Collart et al., 2013; Collart et al., 2017), histone proteins (Amodeo et al., 2015; Chari et al., 2019; Shindo and Amodeo, 2019; Shindo and Amodeo, 2021) and dNTPs (Vastag et al., 2011; Djabrayan et al., 2019; Liu et al., 2019) (Figure 2A). Decreased availability of these factors may cause DNA replication stress, leading to S-phase elongation via activating Chk1 (Collart et al., 2013; Chari et al., 2019). Independent of its effect on Cdk1, Chk1 was shown to elongate S-phase through degradation of the replication factor Drf1 in *Xenopus* embryos (Collart et al., 2017). Furthermore, it was recently shown that *Drosophila* histone proteins can act directly as competitive inhibitors of Chk1 and thus prevent cell cycle slowdown independently of their incorporation into the chromatin (Shindo and Amodeo, 2021).

In the cytoplasm maternally provided RNA and protein of the cell cycle regulators Cdk1, Cdc25, Chk1 and cyclins dictate cell cycle progression [reviewed in (Heim et al., 2017; Zhang et al., 2017; Brantley and Di Talia, 2021)]. Their activity is regulated translationally and post-translationally (Heim et al., 2017; Zhang et al., 2017). Maternally provided are also metabolites, such as dNTPs [reviewed in (Liu and Großhans, 2019)], and reservoirs like lipid droplets (Dutta and Sinha, 2017; Kilwein et al., 2023) and yolk platelets (Shimogama et al., 2022) that supply energy and building blocks for cell cycle progression during cleavages (Figure 2A). Last, the nuclear and cytoplasmic compartment may also interact mechanically, such as in *Xenopus* extracts, where yolk platelets have been recently shown to impede nuclear expansion, which could affect nuclear import and thus DNA replication (Shimogama et al., 2022).

All in all, the N/C ratio appears as a common coarse-grained controller of cell cycle lengthening, reflecting changes in the relative concentration of several molecular factors in the nucleus and/or cytoplasm. Cell-to-cell differences in N/C ratio appear to play an essential role in establishing cell cycle heterogeneity within a population (see Section 2.2), and as a result impact collective tissue properties during development (see Section 2.3).

#### 2.1.2 Zygotic transcription

At the MBT, some species also undergo large-scale activation of the zygotic genome (ZGA), enabling the transition from maternal to zygotic control (Tadros and Lipshitz, 2009). This temporal correlation may be causal, since transcription may be required for cell cycle progression itself, due to the synthesis of cell cycle regulators (Zhang et al., 2017) (Figure 2C). For instance, in *Drosophila*, it was found that zygotic transcription enables cell cycle lengthening at MBT through at least two mechanisms [reviewed in (Liu and Großhans, 2019)]: (i) Large-scale ZGA increases S-phase through activation of the DNA replication checkpoint via Chk1 (Blythe and Wieschaus, 2015); (ii) Products of zygotic transcription decrease Cdc25 activity, a positive regulator of Cdk1, increasing the G2-phase of the cell cycle (Farrell et al., 2012; Di Talia et al., 2013; Farrell and O’Farrell, 2013). Furthermore, zygotic transcription also plays a role during cell fate acquisition, which can in turn control cell cycle length (see section 2.2). In contrast, inhibition of zygotic genome activation in zebrafish only partially impacts cell cycle lengthening (Kane et al., 1996; Zhang et al., 2015). Given that ZGA and cell cycle remodeling may be both dependent on changes in the N/C ratio (Balachandra et al., 2022) and that the cell cycle itself may control ZGA as well (Schulz and Harrison, 2019; Strong et al., 2020), it is hard to decipher the dependency between the two. For example, transcription takes time and cannot take place during mitosis, so longer cycles are needed for synthesizing more and longer mRNA transcripts (Rothe et al., 1992; Schulz and Harrison, 2019; Vastenhouw et al., 2019; Strong et al., 2020) (Figure 2C). In addition, at the start of development, the small amount of DNA template that is mostly occupied by histone proteins might also limit transcription sterically (Vastenhouw et al., 2019). As a result, several lines of evidence suggest that transcription and cell cycle lengthening are functionally dependent, however, their dependency may rely on species-specific developmental programs.

#### 2.1.3 Maternal mRNA regulation

Oocytes are preloaded with maternal mRNA and protein that determine the cell cycle length until zygotic transcription takes over (Murray and Kirschner, 1989; Tadros and Lipshitz, 2009). mRNA translation is regulated both within each cycle and also throughout early development to change from maternally supplied to zygotic control. In every cell cycle, cyclin proteins degrade and thus, prior to zygotic transcription, the embryo solely depends on translation of maternal mRNA for cell cycle progression (Heim et al., 2017). Additional cell cycle proteins are regulated by mRNA levels, such as maternal Cdc25 in *Drosophila* (Edgar and Datar, 1996). During embryo development, translation and degradation of maternal mRNAs can be controlled by polyadenylation, RNA modifications, RNA binding proteins, and microRNAs [reviewed in (Vastenhouw et al., 2019)]. Factors actively controlling these processes may be maternally provided, like SMAUG in *Drosophila* (Tadros et al., 2007), or products of zygotic transcription, like the microRNA miR430 in zebrafish (Giraldez et al., 2006). Here we will briefly mention some examples of how mRNA regulation can affect cell cycle remodeling in early embryos.

Translation and degradation of mRNAs can be regulated by the length of their polyA tail (Figure 2D). Before MBT, in *Xenopus*, zebrafish and *Drosophila* embryos, translational efficiency positively correlates with polyA tail length. After MBT, short poly-A tails seem to not affect translation-efficiency, but lead to mRNA degradation (Subtelny et al., 2014; Eichhorn et al., 2016). Fundamental work in *Xenopus* extracts has shown that cell cycle progression is driven by polyA-dependent translation of maternal CyclinB mRNA (Stebbins-Boaz et al., 1999; Groisman et al., 2002; Cao et al., 2006). mRNA polyadenylation changes drastically around MBT in *Xenopus and* zebrafish, showing decreased polyadenylation for mitosis-associated transcripts and increased polyadenylation for DNA damage checkpoint associated transcripts (Collart et al., 2014). Polyadenylation is enabled by cytoplasmic polyadenylation-element binding proteins (CPEBP) that bind to the 3′UTR of mRNAs in early embryos of *Xenopus*, zebrafish and mouse (Collart et al., 2014; Sha et al., 2017; Winata et al., 2018) (Figure 2D). To remove the polyA tail, different RNA binding proteins recruit the de-adenylation machinery enabling clearance of maternal mRNAs during the maternal-to-zygotic transition across various species [reviewed in (Vastenhouw et al., 2019)].

RNA modifications can also impact mRNA lifetime and translation efficiency. For example, adenine methylation (N6-methyladenosine - m6A) may increase translation efficiency or promote degradation, depending on the protein that binds it (Wang et al., 2015). In zebrafish, mRNA clearance and cell cycle progression following MBT were shown to be impacted by m6A and its recognition by a m6A-binding protein (Zhao et al., 2017) (Figure 2D). Similarly, in mice, knockdown of a m6A writer suggested a role for m6A-driven mRNA translation and degradation of cell cycle regulating transcripts during the first cell cycle (Sui et al., 2020). Additionally to the protein-coding function of maternal RNAs, it was suggested that their degradation may act as a source for dNTP synthesis required for genome duplication (Vastenhouw et al., 2019), which could enable S-phase progression upon depletion of maternal dNTP pools (Figures 2A,D). Thus, the selective temporal and spatial regulation of maternal mRNAs is an essential regulator of cell cycle length.

Altogether, the above mechanisms explain how cells lengthen their cell cycles, however, why each cell lengthens differentially its cycle leading to desynchronization within a cell population is less understood. Elucidating the mechanisms of the emergence of variability in cell cycle lengths is key for addressing the collective effects of cell cleavage dynamics in embryogenesis. Below we describe mechanisms underlying differential cell cycle lengthening between embryonic cells.

### 2.2 Mechanisms underlying cell-to-cell cycle length variability

Variability in cell cycle lengths, or (de)synchronization, has been suggested to be the outcome of deterministic and/or stochastic processes. For instance, inherent differences within the embryo, such as pre-existing patterns in gene expression that affect the concentration of the molecular components of the cell cycle machinery or pre-existing differences in the cell size that impact the N/C ratio can act as deterministic regulators of cell-to-cell cycle length variability. In contrast, cell cycle length variability can also be an outcome of stochastic self-organizing processes, for example, via the emergence of mitotic waves from the auto-regulatory feedback loops of the cell cycle oscillator and their intercellular diffusion (Fuller, 2010).

#### 2.2.1 Self-organized cell cycle variability

In a syncytial system, nuclei distribution can regulate mitotic cycle synchronicity via self-organization. This can result in meta-synchronous mitotic waves propagating through the cell, as is the case in the early *Drosophila* embryo [reviewed in (Brantley and Di Talia, 2021; Liu et al., 2021; Lv et al., 2021; Padilla et al., 2022)]. In *Drosophila*, the three first rounds of division occur in the center of the blastoderm. During rounds four to six, nuclei spread along the anterior-posterior (A-P) axis in a process called “axial expansion”. During rounds seven to nine, nuclei migrate to the embryo surface where they continue with the last four meta-synchronous divisions before eventually cellularizing and forming an epithelium (Zalokar and Erk, 1976; Foe and Alberts, 1983; Baker et al., 1993; Lv et al., 2021; Padilla et al., 2022). Meta-synchrony is observed with the nuclei at the anterior and posterior poles dividing first and in the middle later (Foe and Alberts, 1983). The uniform spatial organization of the nuclei along the A-P axis was shown to ensure uniform N/C ratio, which initiates cell cycle elongation at MBT and thus maintains cell cycle synchronization (Deneke et al., 2019). Intriguingly, the uniform nuclei distribution is controlled by the cell cycle oscillators themselves that control actomyosin contractility (see Section 2.3), but also by the microtubule asters (de-Carvalho et al., 2022). In this system, the sensing of the N/C ratio has been shown to be collective, where nuclei within a ∼100 μm radius display the same cell cycle dynamics (Hayden et al., 2022).

**FIGURE 3:**
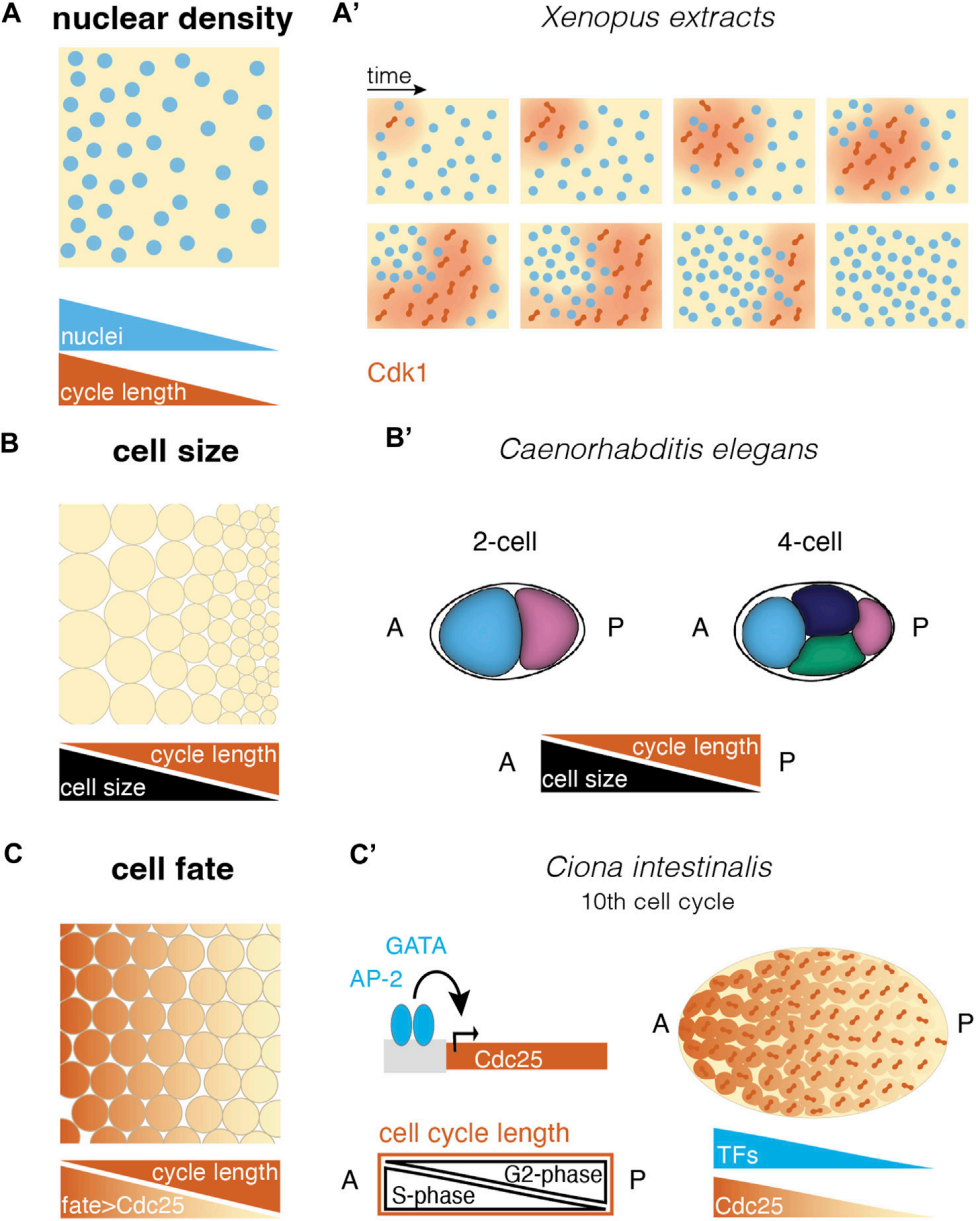
Different ways to create cell cycle differences in a multinucleated or multicellular system. **(A)** Self-organized cell cycle duration and mitotic entry via local nuclear density and an example in the (**A′**) *Xenopus* extract system. Cdk1 diffusion upon nuclear envelope breakdown (red) triggers mitotic entry starting from a pacemaker nucleus in a low nucleus density region (after (Chang and Ferrell, 2013; Nolet et al., 2020)). **(B)** Cell size correlates negatively with cell cycle length in different species. This allows asymmetric divisions to set up cell cycle length differences in a tissue (**B′**) e.g., in the *C. elegans* embryo, asymmetric divisions lead to larger anterior cells with shorter cell cycles, resulting in a division wave throughout the embryo (Deppe et al., 1978). **(C)** Fate-dependent transcription factors may control the expression of cell cycle regulators, resulting in cell cycle length regulation and mitotic domains according to cell differentiation. (**C′**) In the ascidian *Ciona intestinalis*, the transcription factors GATA and AP-2 have been suggested to control Cdc25 expression along the anterior-posterior axis. This causes a gradient in G2-phase that compensates for a S-phase gradient, leading to equal cell cycle length throughout the embryo and mitotic synchrony (Ogura et al., 2011; Ogura and Sasakura, 2017). [abbreviations: A anterior; P—posterior; TFs: transcription factors].

How exactly adjacent nuclei affect each other’s division cycle is still a subject of active research. *In vitro* work in *Xenopus* extracts suggested that the mitotic waves are the outcome of chemical waves of Cdk1 activity (Chang and Ferrell, 2013). As nuclei approach mitosis they elevate Cdk1, which is released upon nuclear envelope breakdown. Thus, active Cdk1 molecules can diffuse to neighboring nuclei. Together with positive feedback (Figure 2A, inset), Cdk1 quickly increases in the new region, triggering mitotic entry, propagating as a trigger wave across long distances (Chang and Ferrell, 2013). Here, it was further shown that waves originate at regions of less concentrated nuclei acting as pacemakers, with higher nucleus concentration leading to slower waves. Lower nuclei concentration seems to result in less competition for and higher nuclear import of cell cycle regulators, making a nucleus in a less dense region faster (Figure 3A). This nucleus can then trigger the auto-amplification loop controlling the wave propagation (Afanzar et al., 2020; Nolet et al., 2020) (Figure 3A’). However, the spread of the mitotic waves observed in the syncytial *Drosophila* blastoderm cannot be solely explained by trigger waves (Deneke et al., 2016). In this system, during division 10–13, cell cycles slow down due to an elongation of the S phase (Farrell and O’Farrell, 2014) and activation of the DNA-replication checkpoint (Fogarty et al., 1997; Sibon et al., 1997; Farrell and O’Farrell, 2014), with the latter controlling Cdk1 through the Chk1/Wee1 pathway. By imaging both Cdk1 and Chk1 activity, it was demonstrated that the slowing down of the Cdk1 waves is not due to a slower activation of the mitotic switch but because of the Chk1 activity becoming higher. As a result, a longer time is required to overcome the inhibition of Cdk1 by Chk1, leading to a slowing down of the propagation of the Cdk1 activity (Deneke et al., 2016). This separates the S-phase trigger waves from those happening at the M-phase, which was characterized as a purely kinematic process, or a sweep wave. Similarly, crosstalk of trigger and sweep waves has been observed later on in *Drosophila* and *Xenopus* extracts, when the cell cycle slows down, potentially upon depletion of resources in the system (Vergassola et al., 2018; Nolet et al., 2020; Puls et al., 2024).

The above self-organizing pathways of cell cycle (de)synchronization have been described in systems that share cytoplasm. However, meta-synchronous mitotic waves have been also observed before MBT in systems with cell boundaries, such as *Xenopus* and zebrafish, and they are typically directed from the animal to the vegetal pole (Newport and Kirschner, 1982; Kane and Kimmel, 1993; Kimmel et al., 1995; Keller et al., 2008; Olivier et al., 2010; Anderson et al., 2017). By imposing a temperature gradient in the *Xenopus* early embryo, it was shown that the cold and warm sides of the embryo could continue their periodicity independently, suggesting that mitotic waves originate from cell-autonomous clocks that are ticking at different rates and there is no cell-cell communication in regulating the embryo-scale mitotic waves (Anderson et al., 2017). Similar animal-vegetal oriented mitotic waves have been observed in zebrafish (Keller et al., 2008; Olivier et al., 2010), however, if in systems with cell boundaries mitotic waves are self-organized or the consequence of some pre-patterning factor is yet unclear.

#### 2.2.2 Pre-existing cell size inequalities

In several species an inverse correlation between cell cycle duration and the cell size is observed upon MBT. One of the most well-characterized examples of how cell size triggers cell cycle desynchronization has been described in *Xenopus*. Due to the inherent polarity of the *Xenopus* zygote where the vegetal pole has most of the yolk platelets, vegetal pole cell divisions take longer than those at the animal pole due to higher resistance of the closing of the cleavage furrow (Gilbert, 1985; McDougall et al., 2019). As a result, a cell size gradient is set along the animal-vegetal axis (Chalmers et al., 2003; Strauss et al., 2006). Intriguingly, cell size is irrelevant for cell cycle length before MBT (Wang et al., 2000). Once the cells cross the critical “coarse-grained” factor, the N/C ratio, they start increasing the duration of the S phase based on their size (Iwao et al., 2005), and since the early blastomeres exhibit large size inequality (Figure 3B), this correlation is sufficient to explain cell division asynchrony in this model.

Cell size inequalities also regulate division desynchronization in zebrafish upon MBT (Kane and Kimmel, 1993; Kimmel et al., 1995; Keller et al., 2008; Olivier et al., 2010) with indications that such inequalities can be inherited to the daughter cells (Kane and Kimmel, 1993; Kimmel et al., 1995; Keller et al., 2008; Olivier et al., 2010). In this system however, additional regulators of cell cycle length are the maternal RNA degradation (Zhao et al., 2017), and presumably ZGA (Dalle Nogare et al., 2009; Zhang et al., 2015). Some echinoderms also show a strong cell size dependence of the cell cycle length (Emura and Yajima, 2022). For example, the embryos of the sand dollar show a mitotic gradient due to a cell size gradient. Here, cell cycles become asynchronous upon hatching, correlating with changes in protein synthesis, respiration and transcriptional activity prior to gastrulation (Duncan and Whiteley, 2011).

Cell size inequalities might be important in certain species irrespective of MBT. For instance, in *C. elegans* embryos due to asymmetric PAR activity in the zygote, the mitotic spindle is positioned asymmetrically, giving rise to smaller cells at the posterior and thus partitioning the cytoplasmic fate determinants (Deppe et al., 1978; Kemphues et al., 1988; Rose and Gönczy, 2014). These cells divide later than the anterior cells, generating an anterior-posterior mitotic wave along the embryo (Figure 3B’). Intriguingly, in *C. elegans*, where cells are smaller to begin with and there is no MBT (Schauer and Wood, 1990), the cell cycle length dependency on cell size is evident from the first division. Furthermore, in medaka, cell cycle desynchronization also occurs before MBT, however, its origin might be due to the combined effects of both cell size inequalities emerging from highly asymmetric cell cleavages and the early activation of the zygotic genome (Kraeussling et al., 2011). Last, cell size asymmetries might also exist in the zebrafish early cleavages due to asymmetric centrosome positioning, but their effects on cell cycle length before MBT remains to be elucidated (Rathbun et al., 2020).

#### 2.2.3 Cell fate determinants and pre-patterning

In contrast to the above examples, there are species where the differential cell cycle lengthening is independent of cell size. In ascidians for instance, the first cell cleavages are synchronized despite the blastoderm cells exhibiting big differences in their cell sizes (Tassy et al., 2006; Dumollard et al., 2017; Godard et al., 2021). Upon MBT, which occurs at the 16-cell stage (Dumollard et al., 2013), asynchronous mitosis is observed that coincides with the specification of the endo-mesoderm (Imai et al., 2000). β-catenin expression was shown to accelerate the S-phase leading to faster divisions in the vegetal blastomeres suggesting that cell fate cues overrule the impact of cell size in cell cycle lengthening, and in this case directly regulates mitotic desynchronization (Dumollard et al., 2013).

Along the same lines, certain cell fates were shown to display unique cell cycle clocks, leading to the generation of mitotic domains, and thus embryo-scale asynchrony. Indeed, several species exhibit mitotic domains including *C. elegans*, ascidians, *Drosophila* and zebrafish (Deppe et al., 1978; Nishida, 1986; Foe, 1989; Kane et al., 1992), presumably arising from fate-dependent regulation of a set of transcription factors that directly impact cell cycle duration (Rothbächer et al., 2007; Ogura and Sasakura, 2016; Sasakura et al., 2016; Imai et al., 2017). In *Drosophila*, 25 mitotic domains were mapped, where their differential cell division timings during divisions 14–16 (after MBT) depend on the transcriptional activation of *string*, which is one of the two cell cycle regulators Cdc25 phosphatases (Edgar and O’Farrell, 1989; Foe, 1989; Edgar and O’Farrell, 1990; Edgar et al., 1994) (Figure 3C). Cdc25 expression is regulated by patterning genes controlling cell fate specification explaining the differential timing of cell divisions between the mitotic domains (Edgar et al., 1994). Furthermore, within a mitotic domain a subset of activators and repressions of Cdc25 transcription is tuning division timing (Momen-Roknabadi et al., 2016). Another example of the generation of mitotic domains comes from ascidians where the S-phase of the 11th cell cleavage is significantly longer than the previous cycles generating a meta-synchronous posterior-to-anterior mitotic wave at the epidermis (Ogura et al., 2011) partitioned in three distinct mitotic domains and a bidirectional wave in a fourth domain. Intriguingly, in the previous division the long S-phase observed on the anterior side is compensated by a shorter G2-phase leading to mitotic synchrony (Figure 3C’). This compensation mechanism is enabled by the downregulation of the expression of Cdc25 at the anterior side, upon downregulation of patterning genes GATA and AP-2 at the onset of neurulation (Ogura and Sasakura, 2016) (Figure 3C’).

Last, in *C. elegans*, where although there is a strong dependency on cell size, each lineage displays different strength of this correlation (Arata et al., 2014). Already from the 2-cell stage, PAR proteins concentrate more Polo kinase PLK-1 (Budirahardja and Gönczy, 2008; Rivers et al., 2008), and factors of the replication machinery to the anterior larger cell (Gaggioli et al., 2020) that can impact cell cycle lengths.

Altogether, both deterministic and stochastic processes underlie the emergence of spatial and temporal variations in cell cycle length. If and how such modes of regulation act together during development to tightly control the degree of cell cycle variation in the developing embryo remains to be addressed.

### 2.3 Collective effects of cell cleavage dynamics

Cell cleavage (de)synchronization and their dynamics were shown to impact processes occurring either further away from where division takes place, such as the cell cortex, or at a global scale, such as properties emerging from the coordination of many cells including tissue shape, fate and physical characteristics. How cleavage dynamics influence collective behavior has only recently started to be explored. Below we highlight work on this topic, by categorizing it in systems with shared cytoplasm, e.g., syncytia or early embryos, and in systems composed of cell compartments, e.g., multicellular structures.

#### 2.3.1 Collective effects in systems with shared cytoplasm

**FIGURE 4:**
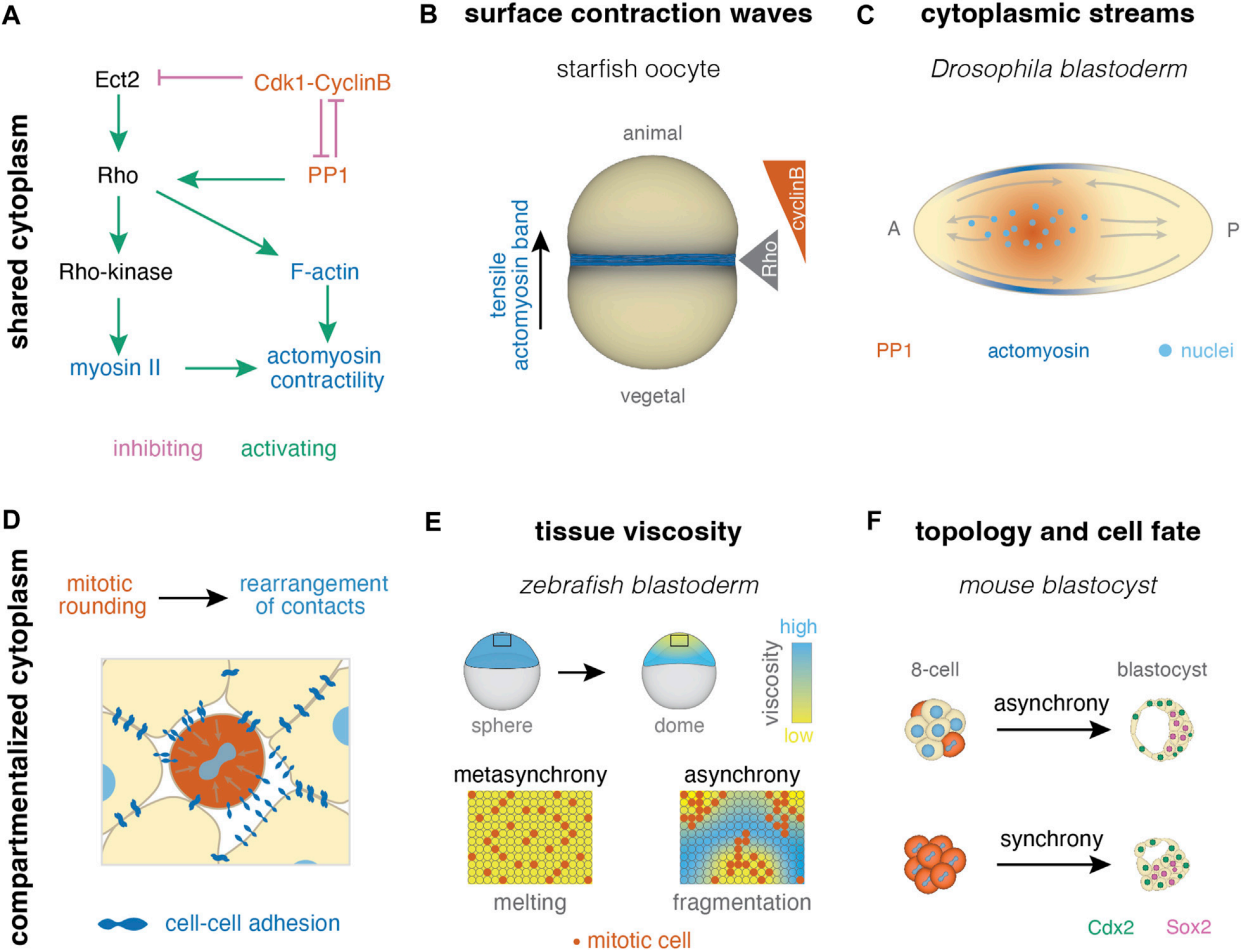
Collective effects of cell cleavage dynamics in systems with shared and compartmentalized cytoplasm. **(A)** In systems with shared cytoplasm, spatial patterns of contractility can be induced by pathways linking the cell cycle and actomyosin skeleton (pathway after (Bement et al., 2015; Basant and Glotzer, 2018; Deneke et al., 2019)). **(B)** For example, surface contraction waves are regulated by Cdk1-cyclinB dynamics in starfish oocytes (Bischof et al., 2017) **(C)** Cytoplasmic streams can result from PP1 induced cortical contractility upon its release from inhibition by Cdk1 at mitosis exit, as seen during early divisions in *Drosophila* (Deneke et al., 2019). **(D)** In multicellular systems, cell divisions cause rearrangement of cell-cell contacts due to mitotic rounding. **(E)** Homogeneous distribution of mitotic rounding enables uniform tissue fluidization (melting) in the zebrafish blastula (Petridou et al., 2019; 2021). **(F)** Mitotic synchrony has also been suggested to affect cell packing and downstream cellular fate in the early mouse embryo (Fabrèges et al., 2023).

Cell cycle regulators can impact cytoskeletal components, usually to drive cell division (Bement et al., 2015; Basant and Glotzer, 2018) (Figure 4A). In developing systems with shared cytoplasm, cell cleavage dynamics can thus coordinate embryo scale collective behavior by directly impacting properties of the actin cytoskeleton, including polymerization and contractility that can travel through long distances as a wave (Figure 4A). The very first divisions in *Xenopus*, *Drosophila* and starfish are often accompanied by cell cycle-dependent cortical contractility, or surface contraction waves (SCWs) (Rankin and Kirschner, 1997; Royou et al., 2002; Bement et al., 2015). For instance, in *Xenopus* and zebrafish oocytes contraction waves are observed traveling from the animal to the vegetal pole driven by the wave-like activation and inactivation of Cdk1 (Rankin and Kirschner, 1997; Chang and Ferrell, 2013; Shamipour et al., 2019). During starfish oogenesis, due to an initial gradient of Cdk1 activity originating from the asymmetrically located nucleus, a wave of contractility is observed. Contractility is activated by removal of Cdk1 inhibition of the RhoA/RhoA kinase/Myosin II signaling module and it is switched off by negative feedback downstream of RhoA kinase itself (Bischof et al., 2017) (Figures 4A, B). Such waves spanning the whole zygote were shown to be important for cytoplasmic organization.

For example, in *Drosophila*, the intricate relationship between the cell cycle and contractility was shown to drive nuclei positioning, during the process of axial expansion. During this time, the nuclei are positioned in the middle of the embryo, where local downregulation of Cdk1 at mitotic exit triggers a damped spreading of PP1 activity, a mitotic phosphatase, reaching to ∼40 μm away from the nuclei. The latter recruits myosin II, likely via Rho, at the cortex surrounding the nuclei, generating gradients of contractility. The cortical contractions at the middle of the embryo result in mid-embryo cytoplasmic flows that push the nuclei towards the poles, and thus driving axial expansion (Deneke et al., 2019) (Figure 4C). As mentioned earlier, the correct positioning of the nuclei is in turn required to keep cell cycle synchronization (see Section 2.2). In the later stages of *Drosophila* development, cycles 12–13, the nuclei exhibit a yo-yo-like motion during the meta-synchronous mitotic wave (Lv et al., 2020), where nuclei move collectively and anisotropically over several nuclei diameters away from the mitotic wave front, and then return back to their original position. It was recently shown that this nuclei movement is driven by two components: (i) the degree of elongation of the isotropically oriented mitotic spindles occurring during anaphase, and (ii) cortical F-actin bringing back the nuclei to their initial position. Interestingly, the degree of elongation of the mitotic spindles was theoretically predicted to be regulated by the meta-synchronous nucleus cycles *per se* (Lv et al., 2020). Following cleavage cycles, the *Drosophila* embryo undergoes cellularization (Lecuit and Wieschaus, 2000) [reviewed in (Schmidt and Grosshans, 2018; McCartney and Dudin, 2023; Sokac et al., 2023)]. Here, it has been shown that membrane furrows for cellularization are positioned at metaphase furrows of the previous nuclear cycle (He et al., 2016). In the insect *Tribolium castaneum*, cleavage furrows not only from one previous division cycle, but multiple, are used as locations for cellularization (van der Zee et al., 2015). Overall, this suggests that the cleavage dynamical pattern in the syncytium impacts tissue architecture for downstream developmental processes.

Another form of cytoplasmic reorganization driven by cell cleavage dynamics is the ooplasmic segregation in the zebrafish early embryo, during its first cell cleavages (Shamipour et al., 2019). The zebrafish oocyte is a mixture of yolk granules and ooplasm, which starts to separate after fertilization (Beams et al., 1985; Leung et al., 2000). Ooplasm flow to the animal side of the embryo is driven by cell cycle-mediated bursts of bulk actin polymerization, and not cortical contractility, which proceeds as a wave throughout the large zygote, with a period dictated by Cdk1 activity. Since the first cell cleavages in zebrafish share cytoplasmic components (Kimmel and Law, 1985), their synchronicity coordinates actin polymerization at the embryo-scale (Field et al., 2011; Shamipour et al., 2019). Last, it is worth mentioning that recent work in self-organized *Xenopus* extracts has identified another link between cell cleavages and cytoplasmic reorganization, the microtubule cytoskeleton. Although the effects of cell cycle synchronicity in cytoplasmic organization in extracts is yet to be determined, cycling extracts build arrays of astral microtubules that define cell-like compartments, even in the absence of nuclei (Cheng and Ferrell, 2019). Together with more recent work in *Drosophila* syncytium showing that centrosomes in the absence of Cdk/Cyclin activity can drive cytoplasmic divisions leading to extrusion of aberrant nuclei (Bakshi et al., 2023), it raises the hypothesis that microtubules may communicate dividing cues at the collective level.

#### 2.3.2 Collective effects in systems with cell compartments

In developing systems with cell compartments separated by physical boundaries, a common mechanism by which cell cleavages coordinate embryo scale properties is by impacting cell-cell adhesion [reviewed in (Godard and Heisenberg, 2019; McDougall et al., 2019)] (Figure 4D). During cell cleavages, cell-cell contacts undergo remodeling, with different degrees between species, changing their topology (Giammona and Campàs, 2021). This implies that the cell cycle length, the orientation of cell division and the forces between the blastomeres will play an essential role in embryo packing configurations, tissue scale physical properties and patterning.

A recent example of how cell cleavage synchronicity regulates tissue topology was described in zebrafish, where the effects on tissue topology were shown to influence tissue deformability and morphogenesis. In this system, the first morphogenetic movement starts with the process of doming, where the blastoderm starts to spread on top of the yolk cell (Kimmel et al., 1995; Bruce and Heisenberg, 2020). Blastoderm spreading was found to be facilitated by an abrupt drop in tissue viscosity, fluidization, that increases tissue deformability and allows it to spread (Petridou et al., 2019). This study has shown that fluidization is an outcome of cell-cell contact remodeling induced by the fast rounds of cell cleavages, with the fluidization time point corresponding to the last cleavage round (Petridou et al., 2019) (Figure 4E). Pharmacological inhibition of cell division and specific interference with the mitotic rounding occurring during the cell cleavages, revealed that there is a tug-of-war between cell-cell adhesion forces and mitotic rounding forces, with the latter dominating and inducing contact disassembly and tissue fluidization (Petridou et al., 2019). In a follow-up study, it was shown that tissue fluidization is triggered by a rigid-to-floppy phase transition when the blastoderm reduces its connectivity below a critical point, raising the hypothesis that the spatiotemporal program of cell cleavages encodes information of how many contacts per cell should be disassembled to trigger the rheological change. In order for a system to undergo a uniform phase transition, the microscopic components should exhibit random changes in their interactions. This suggests that the location of the cell cleavages within the tissue should be defined at random, in order for the connectivity changes to occur at random and thus, for the tissue to effectively “melt” and fluidize (Petridou et al., 2021) (Figure 4E). This study uncovered that cell cleavage meta-synchrony serves as an effective mechanism for inducing robust fluidization, since experimental induction of cell cleavage asynchrony resulted in incomplete, unstable and fragmented patterns in tissue rheology (Figure 4E). Interestingly, at the last round of cell cleavages in *Drosophila*, that is concomitant with the cellularization process as mentioned above, a fast tissue softening is also observed (D’Angelo et al., 2019) indicating that the end point of cell cleavages may be coupled with drastic rheological changes in several species.

In agreement with this notion, recent work in mammals, ascidians and crustaceans point to the observation that cell cleavage synchronicity can influence tissue rheological and/or ordering properties, but in these cases resulting in opposite effects, e.g., tissue tight packing and rigidification. For instance, the development of ectodermal segments in the crustacean *Paryhale hawaiensis* was shown to rely on the combination of proliferative mitotic waves along the D-V axis with oriented cell divisions, and local unoriented cell cleavages along the A-P axis (Cislo et al., 2023). Oriented cell divisions enable homogeneous distribution of cells in rows before fast, unoriented cell cleavages increase the cell number. Via the coordination of the timing of the proliferating and cleaving domains, cells are orderly distributed, which is key for the following formation of segments in the adult body (Cislo et al., 2023). Similarly, in ascidians, cell division synchronicity is thought to underlie ordered development. Ascidian embryos display an invariant cleavage pattern, where the spindle is positioned along the cell’s long axis in the apical plane. It was found that spindle orientation stems from the inherent asynchrony in cell cleavages, where planar cell divisions between ectoderm and endomesoderm alternate. Abolishing this asynchrony resulted in spindle misorientation, and thus in a disrupted spatial cleavage pattern (Dumollard et al., 2017). This stereotypical pattern of cell cleavages was further shown to restrict contact mixing and rearrangements, since shared contacts were maintained throughout the cell division. Such a feature was shown to be crucial for the first fate decisions, since the stable cell-cell communication is favoring a contact area-dependent induction rather than the formation of morphogen gradients (Guignard et al., 2020). Last, recent evidence from studies in mammalian development suggest that cell cleavage synchronicity underlies both rigid tissue packing and robust patterning. Upon comparing mouse, rabbit and monkey blastocysts, it was identified that cell cleavages desynchronize in a stochastic manner, despite the early embryo displaying a robust 3D structure with fixed proportions of inner cell mass (ICM) and trophectoderm (TE) lineages (Fabrèges et al., 2023). This research has shown that mouse embryos at 8-cell and 16-cell stage progressively change their cellular connectivity to an energetically favored topology driven by the interplay of noise and actomyosin-driven compaction (Figure 4F). This topology favors a configuration of a higher number of outer cells, which was previously shown to be crucial for the fate decision between ICM and TE. Interestingly, synchronization experiments resulted in defective embryo packing, reducing the number of the inner cells, leading to imprecise patterning of Sox2 and Cdx2, key markers of ICM and TE fate respectively (Fabrèges et al., 2023) (Figure 4F). The morphogenetic event of compaction is not only observed in mice, but also in human embryos (Maître et al., 2015; Firmin et al., 2022), with most likely similar regulators, suggesting that the above cleavage-dependent mechanism of embryo shaping might be conserved in mammalian embryos. In fact, recent studies have observed that inherent differences in the degree of desynchronization of the four to eight cell stage cleavage can act as a predictive marker for a successful implantation in both mice and humans (Mashiko et al., 2022), further supporting the notion that cell cleavage dynamics underlie robust development.

Finally, it is worth mentioning that cell cleavage synchronicity can impact cell fate decisions, however if such effects are regulated at the collective level is still unclear. For example, in the sea urchin embryo, cell cleavages in the vegetal micromeres slow down at the 8-cell stage and relocalize β-catenin in the nucleus to differentiate into endoderm and mesoderm (Davidson et al., 1998). In addition, the mitotic domains described above, which are established after cellularization in *Drosophila*, are mapped later in development to specific fates (Edgar et al., 1994; Cambridge et al., 1997). Their division (a)synchrony is thought to facilitate the establishment of cell populations that show similar response to specification signals and avoid conflicts between division and cytoskeletal rearrangements (Grosshans and Wieschaus, 2000). Another example is endoderm specification in *C. elegans*, where due to the earlier initiation of zygotic transcription, the cell cycles are shorter in the endoderm precursor cell which is proposed to impact division orientation, cell migration and gastrulation (Wong et al., 2016). Intriguingly however, in certain cases such as in *Xenopus* and *Drosophila*, desynchronization experiments by imposing temperature gradients, although resulted in imprecise patterning, this effect was only transient (Lucchetta et al., 2005; Anderson et al., 2017), suggesting that compensation mechanisms may exist to correct for the effects of impaired cell cycle synchronicity.

To sum up, the above studies suggest that the timing of cell cleavages and the cell-to-cell variability in cell cycle length impact collective behavior during embryo development, including regulation of long-range cytoskeletal contraction, tissue packing and patterning. However, the underlying cellular mechanisms linking changes in the cell’s microenvironment during division to the global tissue properties are still unclear. Several candidates are increasingly arising, such as the link between CDK/cyclin activity and cell contractility, mitotic rounding-mediated shape changes and cell adhesion remodeling and microtubule cytoskeleton and cytoplasmic organization (Bement et al., 2015; Bischof et al., 2017; Basant and Glotzer, 2018; Cheng and Ferrell, 2019; Deneke et al., 2019; Godard and Heisenberg, 2019; Petridou et al., 2019; Petridou et al., 2021; Bakshi et al., 2023; Fabrèges et al., 2023) (Figures 1, 4). In addition, besides uncovering the biophysical links between cell cycle and collective behavior, specific assays to interfere solely with the synchronicity of the divisions *per se*, and not the cell cycle machinery, are needed to mechanistically understand collective effects of cell cleavage dynamics.

### 2.4 Cell cycle synchronicity beyond early cleavages

In addition to the examples mentioned above, there are indications that the timing and synchronicity of cell cleavages is essential for triggering major morphogenetic transitions. Cellularization for instance, is observed not only in metazoans, but also in holozoa (Lecuit and Wieschaus, 2000; van der Zee et al., 2015; He et al., 2016; Thukral et al., 2022) [reviewed in (Schmidt and Grosshans, 2018; McCartney and Dudin, 2023; Sokac et al., 2023)]. In the case of the Ichtyosporean *S. artica* which develops as a syncytium/coenocyte, it was shown that the N/C ratio, and not the cell size, regulates the timing of cellularization (Ondracka et al., 2018; Olivetta and Dudin, 2023). This constitutes a life cycle transition from a syncytium to a transient multicellular and eventually to a unicellular state. Given that this species can display open or closed mitosis (Shah et al., 2023), it would be interesting to address how the waves of Cdk1 activation can be influenced by the degree of nuclear envelope breakdown. Another process that follows cell cleavages is inversion, observed in the algae Volvox (Höhn and Hallmann, 2011; Höhn et al., 2015; von der Heyde and Hallmann, 2022). Although it is yet unclear what regulates cell cleavage synchronicity and how it impacts inversion, a possible mechanism could be the presence of cytoplasmic bridges connecting these cells (Höhn and Hallmann, 2011; Höhn et al., 2015)), a structure that has been observed in early cleaving embryos as well (Kimmel and Law, 1985; Adar-Levor et al., 2021). Besides cell cleavages synchronicity, cell proliferation spatiotemporal dynamics were also shown to impact major morphogenetic transitions including and not limited to the determination of cell lineages, such as spore differentiation in *Dictyostelium* (Muramoto and Chubb, 2008), and tissue (un-)jamming transitions in the mouse neural tube (Bocanegra-Moreno et al., 2023) and avian primitive streak formation (Firmino et al., 2016).

## 3 Outlook

The synchronicity of cell cycle lengths is implicated in the initiation and/or progression of the morphogenetic program. Although more is known of how each individual cell/nucleus regulates its cycle length, the origin of cell-to-cell cycle length variability and its function in large-scale morphogenetic events is still under investigation.

How does cell-to-cell variability in division times arise within a cell population? Although the cycle length of each individual cell is regulated by intracellular signaling, if its cell-to-cell variability is an outcome of cell autonomous vs. non-autonomous, or deterministic vs. stochastic processes is poorly understood. The advancement of quantitative methods and theoretical modeling in recent years has started to provide insights to the above question (Arata and Takagi, 2019). For instance, statistical analysis of the variance in cell cycle lengths can reveal whether the desynchronization process is a purely stochastic process, e.g., linear increase of the variance in mammals (Fabrèges et al., 2023). Such quantitative approaches can inform if the mechanisms regulating cell cycle (de)synchronization are the outcome of noise, e.g., fluctuations in cell size, RNA production, protein degradation (Elowitz et al., 2002; Blake et al., 2003; Sigal et al., 2006), or of deterministic processes, such as inheritance of cell cycle length and/or size (Sandler et al., 2015; Kuchen et al., 2020), or a combination of both. Given that cell-cell or nucleus-nucleus communication is essential for cell cycle synchronization, the regulators of the coupling mechanisms of the cell cycle oscillators could define the dynamics of cell-to-cell variability in cell cycle length. For instance, systems with shared cytoplasm appear to be more synchronous given the free diffusion of the components of the cell cycle machinery, when compared with systems with isolated cell compartments. In fact, recent theoretical work proposes that in cell networks, where each cell is considered as an oscillator and after a cycle it stays connected to its daughter cell, the cell cycle oscillations at the edges of the network are coupled via diffusion, recapitulating networks observed in several invertebrate species (Smart et al., 2023). It is worth mentioning however, that fungal syncytia differ from other syncytia, with their nuclei divisions being asynchronous, suggesting that some form of cytoplasmic organization impacts nucleus-nucleus communication in this case (Roberts and Gladfelter, 2015). Last, incorporating cues from global tissue architecture can also be useful to comprehend cell cycle dynamics, given that mechanical feedback from either tissue properties or extracellular compartments could impact the entry to mitosis during cell cleavages (Borne and Weiss, 2023; Malmi-Kakkada et al., 2023).

Does the degree of cell variability in cycle length matter? Is there an optimum cell cycle variability for the system? This is a likely scenario since several reports have shown that variability in cell proliferative growth can ensure correct cell type proportions and correct organ size and shape (Hong et al., 2016; Jones et al., 2017; Gruenheit et al., 2018; Le Gloanec et al., 2022). More recent work has pointed out that the same may stand true for an optimum variability in cell cleavage cycles lengths ensuring robust tissue physical properties in mice and zebrafish (Petridou et al., 2021; Fabrèges et al., 2023), signaling and differentiation in mice (Pokrass et al., 2020), and overall development in *C. elegans* (Jankele et al., 2021). Potential mechanisms of how a certain level of cell cycle length variability regulates the above processes relies on the fact that the above variability will trigger heterogeneities in other cell properties, e.g., cell size, shape, and mechanics, that could act as a source of robustness (Figure 1C). It is shown that cell cleavages decrease cell size down to a physiological optimum for development and having the correct cell size and its heterogeneity in a population is fundamental for several developmental processes (Cadart et al., 2019; Fung and Bergmann, 2023). For instance, small cells were proposed to generate more precise morphogen gradients (Adelmann et al., 2023), whereas strong cell size inequalities can lead to aberrant cell distribution within a tissue (Ramanathan et al., 2019), supporting the notion that a fine-tuned degree of cell-to-cell variability might be required for robust developmental progression. This suggests that similarly to microbial communities where heterogeneities in division/growth dynamics are demonstrated to increase fitness (Cerulus et al., 2016; Levy, 2016), in the case of early embryonic development as well, cell cleavage dynamics may be optimally regulated to ensure robust morphogenesis. The development of methodology to quantitatively map temporal variability, e.g., sensitive Cdk1 sensors (Maryu and Yang, 2022), and to specifically interfere with cell cycle variability and not cell cycle length, e.g., via entrainment (Lu and Cross, 2010; Gérard and Goldbeter, 2012) can allow a direct exploration of the above hypothesis.

In conclusion, the above studies suggest that the level of heterogeneity in the lengths of cell cleavage cycles underlies robust development. Although the notion that embryonic trajectories implicate phases of high heterogeneity or disorder to achieve robust phenotypes is counterintuitive, variability can in fact increase information transmission in a system, by allowing a wider dynamic range of responses to the underlying stimuli (Wada et al., 2021). Revealing the mechanisms linking cell cycle variability to embryo-scale coordination will increase our understanding of morphogenetic robustness, and more broadly, of how biological systems transmit information to achieve long range coordination.
